# Did Saving Mothers, Giving Life Expand Timely Access to Lifesaving Care in Uganda? A Spatial District-Level Analysis of Travel Time to Emergency Obstetric and Newborn Care

**DOI:** 10.9745/GHSP-D-18-00366

**Published:** 2019-03-11

**Authors:** Michelle M. Schmitz, Florina Serbanescu, Vincent Kamara, Joan Marie Kraft, Marc Cunningham, Gregory Opio, Patrick Komakech, Claudia Morrissey Conlon, Mary M. Goodwin

**Affiliations:** a Division of Reproductive Health, U.S. Centers for Disease Control and Prevention, Atlanta, GA, USA.; b Baylor College of Medicine Children's Foundation–Uganda, Kampala, Uganda.; c Bureau for Global Health, U.S. Agency for International Development, Washington, DC, USA.; d Infectious Diseases Institute, Makerere University, Kibaale, Uganda.; e Division of Global HIV and TB, U.S. Centers for Disease Control and Prevention, Kampala, Uganda.

## Abstract

**Introduction:**

Interventions for the Saving Mothers, Giving Life (SMGL) initiative aimed to ensure all pregnant women in SMGL-supported districts have timely access to emergency obstetric and newborn care (EmONC). Spatial travel-time analyses provide a visualization of changes in timely access.

**Methods:**

We compared travel-time estimates to EmONC health facilities in SMGL-supported districts in western Uganda in 2012, 2013, and 2016. To examine EmONC access, we analyzed a categorical variable of travel-time duration in 30-minute increments. Data sources included health facility assessments, geographic coordinates of EmONC facilities, geolocated population estimates of women of reproductive age (WRA), and other road network and geographic sources.

**Results:**

The number of EmONC facilities almost tripled between 2012 and 2016, increasing geographic access to EmONC. Estimated travel time to EmONC facilities declined significantly during the 5-year period. The proportion of WRA able to access any EmONC and comprehensive EmONC (CEmONC) facility within 2 hours by motorcycle increased by 18% (from 61.3% to 72.1%, *P* < .01) and 37% (from 51.1% to 69.8%, *P* < .01), respectively from baseline to 2016. Similar increases occurred among WRA accessing EmONC and CEmONC respectively if 4-wheeled vehicles (14% and 31% increase, *P* < .01) could be used. Increases in timely access were also substantial for nonmotorized transportation such as walking and/or bicycling.

**Conclusions:**

Largely due to the SMGL-supported expansion of EmONC capability, timely access to EmONC significantly improved. Our analysis developed a geographic outline of facility accessibility using multiple types of transportation. Spatial travel-time analyses, along with other EmONC indicators, can be used by planners and policy makers to estimate need and target underserved populations to achieve further gains in EmONC accessibility. In addition to increasing the number and geographic distribution of EmONC facilities, complementary efforts to make motorized transportation available are necessary to achieve meaningful increases in EmONC access.

## Abstract (precis)

A spatial analysis of facility accessibility, taking into account road networks and environmental constraints on travel, suggests that the Saving Mothers, Giving Life (SMGL) initiative increased access to emergency obstetric and neonatal care in SMGL-supported districts in Uganda. Spatial travel-time analyses can inform policy and program efforts targeting underserved populations in conjunction with the geographic distribution of maternity services.

## INTRODUCTION

In 2015, an estimated 303,000 women around the world died of a maternal cause, and approximately 201,000 of these deaths occurred in sub-Saharan Africa.^1^ Additionally, almost half of the 2.6 million stillbirths and 30% of newborn deaths in sub-Saharan Africa were due to intrapartum causes.^2^^,^^3^ Most maternal and newborn deaths are preventable with adequate care at birth.^4^ In 2004, the World Health Organization (WHO) recommended skilled birth attendance at every birth and estimated that 50% to 70% of maternal deaths could be averted with timely access to emergency obstetric interventions.^5^^,^^6^ However, access to quality services in low-resource countries continues to be a challenge, especially for women and newborns who require emergency obstetric and newborn care (EmONC).

Physical distance to health care facilities has been widely recognized as an important determinant of accessing health facility delivery.^7^^–^^10^ While EmONC has been considered an essential strategy to save maternal and newborn lives, EmONC coverage in sub-Saharan Africa remains uneven and met need for EmONC has remained low.^11^^–^^13^ For example, in Mali, substantially higher maternal case-fatality rates were associated with travel times greater than 2 hours among women who accessed hospital care in 2005–2007.^14^ For women who need obstetric and other emergency surgery, the benchmark proposed by WHO is no more than 2 hours of travel time to the nearest facility with surgical capacity, which is roughly the interval from onset of bleeding to death if a woman with obstetric hemorrhage does not receive adequate treatment.^15^^,^^16^ Health researchers have suggested that at least 80% of any country's population should have access to selected emergency surgical and anesthesia services, including cesarean deliveries, within the 2-hour time frame.^16^^,^^17^

Adequate availability of EmONC services is defined by WHO as an area having at least 5 EmONC facilities, including at least 1 comprehensive EmONC (CEmONC) facility, per 500,000 population.^15^ Although the ratio of EmONC facilities to the population has been used as a proxy for adequate distance or travel time to reach a facility during an emergency, an optimal geographic distribution of EmONC services is also a critical determinant of timely access.^15^ To achieve the 2030 Sustainable Development Goal 3.1 of reducing global maternal mortality to less than 70 maternal deaths per 100,000 live births,^18^ researchers and policy makers have called specifically for the equitable distribution of EmONC facilities.

Implemented between 2012 and 2017, the Saving Mothers, Giving Life (SMGL) project aimed to rapidly reduce deaths related to pregnancy and childbirth through the implementation of multiple evidence-based approaches to address the 3 dangerous delays pregnant women face in childbirth: delays in deciding to seek care, delays in reaching a facility in time, and delays in receiving quality care at facilities.^19^^–^^22^ To reduce the second and third delays, the SMGL initiative sought to make facility delivery care accessible to all women within 2 hours. This goal required that SMGL-supported districts had a sufficient number of EmONC facilities equitably distributed geographically and adequate transportation to reach appropriate care. Consequently, SMGL-supported efforts in Uganda focused on improving availability and distribution of EmONC services, expanding motorized transportation to these facilities through vouchers for motorcycle taxis, and creating a coordinated ambulance service.^23^^–^^25^

Improved spatial analyses using geographic information system (GIS) technology has expanded our ability to provide more accurate estimates of travel time to and disparities in access to EmONC.^26^^,^^27^ Travel-time modeling, estimating the most efficient travel time to a facility along established roads and walking paths, has emerged as one of the most robust analytical spatial techniques applied in maternal health.^27^ Rather than calculating unrealistic straight-line distances, these algorithms account for the effects of elevation, road conditions, and landscape barriers. Furthermore, these algorithms allow for the estimation of travel times using different transportation modes, such as on foot (walking), bicycle, motorcycle, or car/truck/ambulance (4-wheeled vehicles) and corresponding travel speeds.

GIS has expanded our ability to provide more accurate estimates of travel time to and disparities in access to EmONC.

Because national government health planning is frequently organized and implemented at the district level, spatial analyses to support district and subdistrict interventions are greatly needed. Most studies of accessibility to EmONC care in sub-Saharan Africa, however, have focused on the national level.^28^^–^^30^ A few studies have analyzed point-in-time accessibility at subnational and administrative levels, mapped adverse maternal outcomes, prioritized ambulance services, or pinpointed underserved areas necessitating EmONC upgrades.^30^^–^^32^ This study adds to the literature by using travel-time accessibility modeling to assess changes in estimated travel time to EmONC in SMGL-supported districts in Uganda over the 5-year period of implementation. We examine whether geographic access improved during SMGL implementation and identify areas where access issues persisted at the conclusion of the project.

## METHODS

The 4 SMGL-supported districts in Uganda—Kabarole, Kamwenge, Kibaale, and Kyenjojo—form a contiguous unit in the western region of the country. Among the combined 2017 population of just over 2 million were an estimated 538,706 women of reproductive age (WRA) aged between 15 and 49 years (Table 1).^19^ Population density is low, with over 78% of the 4-district area designated as rural and the largest urban population residing in Kabarole.^34^^,^^35^

**TABLE 1.** Demographic Factors, SMGL-Supported Districts in Uganda, 2016

District	Total Population, 2016^a^	Number of WRA, 2016^a^	Population Density (People/km^2^)^b^	Urbanization Level (% Urban)^b^
Kabarole	456,052	121,794	259	26.0
Kamwenge	392,501	101,650	177	5.5
Kibaale	818,176	206,596	185	7.9
Kyenjojo	428,451	108,666	179	15.4
Total	2,095,180	538,706	173	21.3

Abbreviations: km^2^, kilometers squared; SMGL, Saving Mothers, Giving Life; WRA, women of reproductive age.

a Estimated from SMGL Reproductive Age Mortality Study, 2017.^19^

b 2014 National Census Main Report, Uganda Bureau of Statistics.^33^

**FIGURE 1:**
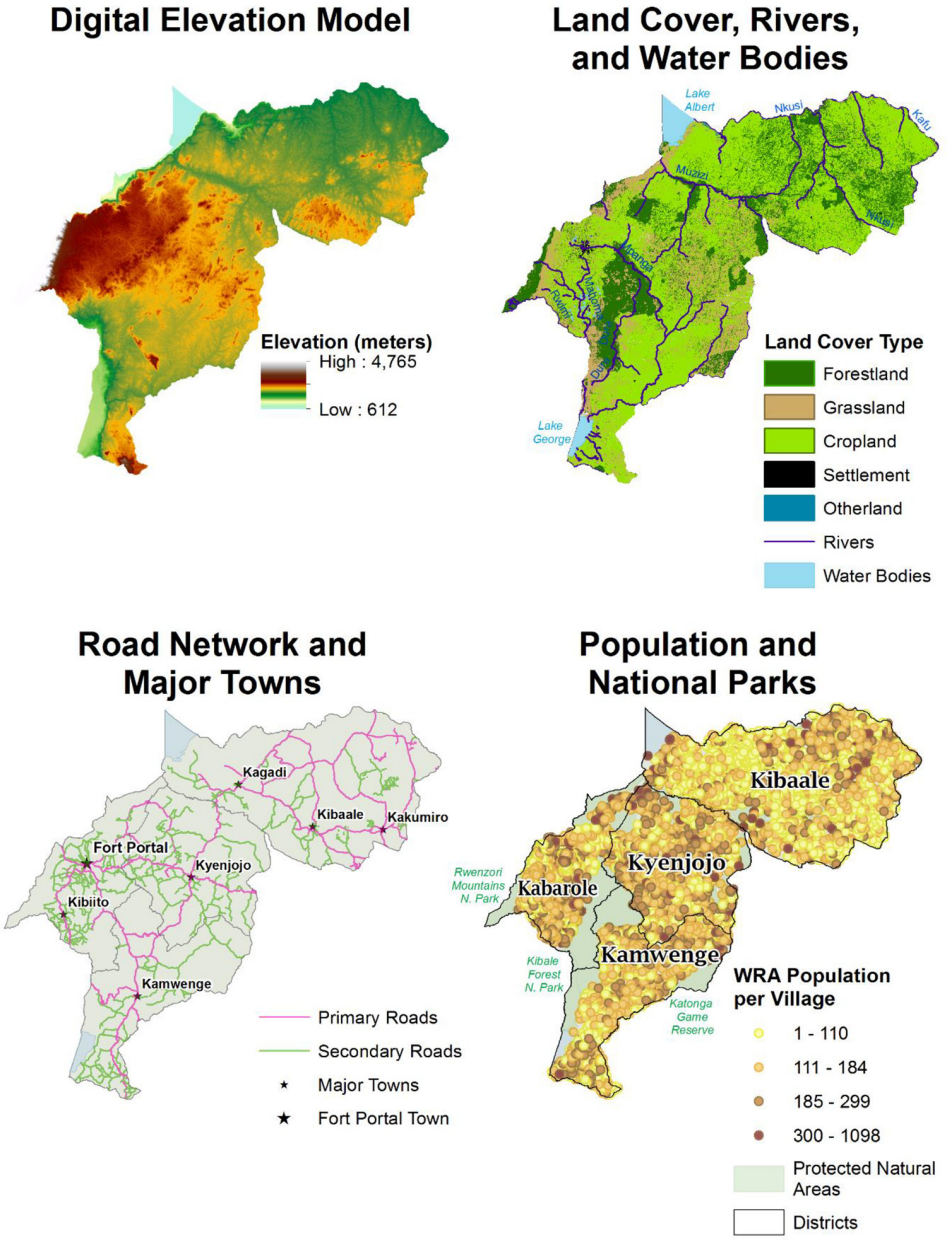
Visual Representation of Data Sources Used in Health Care Accessibility Modeling Analysis Abbreviation: WRA, women of reproductive age.

Transportation challenges are common in the SMGL-supported districts. The topography is mountainous, particularly in Kibaale district. Large national parks are mostly impassable forest and rugged terrain, and numerous rivers and lakes create geographic barriers (Figure 1). Only a small portion of the rural road network is passable by 4-wheeled vehicles, and only 2 paved roads connect Kamwenge and Kyenjojo districts with Fort Portal town, the district capital of Kabarole. Kibaale district did not have any paved roads during the SMGL implementation period.

The measurement of EmONC functionality used in our analysis was based on facility performance of a core set of lifesaving interventions, known as “signal functions,” in the 3 months prior to the health facility assessments (HFAs).^36^ EmONC facilities are defined as having the ability to, at a minimum, (1) administer parenteral antibiotics, (2) administer uterotonic drugs for active management of the third stage of labor and prevention of postpartum hemorrhage, (3) use parenteral anticonvulsants for the management of pre-eclampsia/eclampsia, (4) perform manual removal of placenta, (5) perform removal of retained products, (6) perform assisted vaginal delivery, and (7) perform neonatal resuscitation. CEmONC facilities have the additional capability to perform cesarean deliveries and blood transfusion.^15^ Although the Ugandan Ministry of Health has further mandates about the distribution of government facilities,^37^ our analysis applies WHO benchmarks for EmONC and CEmONC of at least 5 EmONC facilities, including at least 1 CEmONC facility, per 500,000 population. SMGL-supported facilities include those added to the study area during the SMGL initiative as well as existing facilities that were upgraded to provide EmONC.

EmONC functionality was based on facility performance of a core set of life-saving interventions, known as “signal functions.”

### Data Sources

The initiative employed HFAs and other monitoring and evaluation methods to assess the progress and impact of interventions across the SMGL's implementation phases: Phase 0 (pre-implementation planning in 2011–2012), Phase 1 (June 2012 to December 2013), and Phase 2 (January 2014 to October 2017).

#### Health Facility Assessments

To assess changes in facility infrastructure, functionality, and use, SMGL implementing partners in Uganda conducted HFAs in SMGL-supported districts at baseline, the end of Phase 1, and endline (November 2016).^19^ The 3 assessment periods were conducted in 111, 127, and 129 health facilities, respectively, which provided over 95% of all facility deliveries in the SMGL study area at each time point. HFAs documented performance of EmONC functions during the 3 months prior to the assessments as well as the geographic location of facilities (accuracy of ±10 meters).^37^ Our analyses used all facilities included in HFAs for any of the 3 assessment periods.

SMGL-supported health facilities provided care for over 95% of all facility deliveries in the SMGL study area at each time point.

#### Geographic Data

We used land cover data obtained from the Regional Centre for Mapping of Resources for Development,^33^ initially collected with a 30-by-30 meter resolution and subsequently aggregated within AccessMod version 5, revision 5.1.18 (WHO, Geneva, Switzerland,) to a 92-by-92 meter resolution to match the resolution of other layers (Figure 1). This land cover raster used a 6-ecosystem scheme that accounted for forestland, grassland, cropland, settlement, wetlands, and other land cover.

We created updated shapefiles for lakes and rivers using Uganda Bureau of Statistics (UBOS) data^35^ and OpenStreetMap (www.openstreetmap.org) shapefiles. When a data source had incomplete information about a river network, we manually digitized our master river shapefile with Digital Globe EnhancedView Web Hosting Service (https://evwhs.digitalglobe.com/myDigitalGlobe) satellite imagery obtained in June 2018. We considered water bodies as being completely impassable and rivers as being partially passable, if crossed by a primary or secondary road, by an assumed bridge.

We merged and cleaned the shapefiles of the UBOS road network data from the 2014 Uganda Census and the OpenStreetMap road network digitized in mid-December 2017 via the Humanitarian OpenStreetMap Team.^38^^,^^39^ We created subsets of all primary (between district capitals) and secondary road shapefiles (between towns and major villages) for use in the AccessMod analysis (Figure 1). The resulting road shapefile was cross-checked against Digital Globe satellite imagery. We ascertained the proportion of roads that were paved and unpaved and changes in paving that occurred over time. Since the majority of the roads were unpaved and no substantive changes occurred within the project duration, we applied travel speeds for unpaved roads only to yield the most conservative travel time estimates.

Elevation and slope data were obtained from the Shuttle Radar Topography Mission digital elevation model produced by the U.S. Geological Survey, both with a 92-by-92 meter pixel resolution.^40^ The model provided elevation information to the tool and was used to determine the relative slope of each raster pixel.

National parks, from the World Database on Protected Areas, were derived by the United Nations Environment World Conservation Monitoring Centre and considered impassable unless a road passed through it. They were included in the final maps to provide context.^41^

Within AccessMod, the land cover, road network, river, and water body datasets were combined into a merged land cover raster file, with a 92-by-92 meter resolution, and used in the travel-time analyses.

#### Population Data

Household population data from all villages in the 4 Ugandan districts were collected in 2017 as a component of the SMGL Reproductive Age Mortality Study (RAMOS).^19^ While RAMOS's primary aim was to measure and identify main causes of maternal mortality, the study also enumerated households, household members, WRA, and all recent deaths.^19^ We cross-checked geographic coordinates collected in the 2017 RAMOS with UBOS geographic data and reconciled discordant coordinates.^34^^,^^35^^,^^42^ Overall, 538,706 WRA resided in 3,749 villages across the 4 districts in 2016 (Figure 1).

### Analytic Methods

To assess whether districts met the WHO benchmark of EmONC availability, we followed the WHO guidelines, which recommend a minimum of 5 EmONC facilities per 500,000 population, including at least 1 CEmONC facility in each district.^37^ For each district, we calculated the recommended number of EmONC facilities by dividing the estimated district population by 100,000. For each time period, we then computed the observed number of EmONC facilities and compared them to the recommended number of facilities.

We estimated the minimum travel time to the nearest EmONC and CEmONC facilities using the AccessMod Accessibility module. AccessMod uses the least-cost path algorithm to calculate the quickest way of traveling between 2 points, using roads or off-road travel, as appropriate.^43^ Travel time is also dependent on travel speeds for each transportation mode—walking, bicycles, motorcycles, and 4-wheeled vehicles—with land cover influencing the speed of walking. Bicycles and motorcycles can be outfitted with sidecars as makeshift ambulances.^43^^,^^44^ We determined these speeds using direct observation combined with other published sources.^34^^,^^43^^,^^46^^–^^51^ Walking was the only mode of travel used for areas without primary or secondary roads. Speeds were reduced by two-thirds to account for slower transportation speeds of pregnant women and to further account for travel on unpaved roads. Tobler's function, which corrects walking speed based on the direction of slopes on the terrain derived from the digital elevation model, was used to adjust both walking and bicycling speeds.^52^

AccessMod uses the least-cost path algorithm to calculate the quickest way of traveling between 2 points, using roads or off-road travel, as appropriate.

We performed AccessMod travel-time simulations for the 4 transportation modes to EmONC and CEmONC facilities, focusing on a 2-hour upper limit of the estimated travel time, consistent with WHO recommendations for EmONC access.^15^ All estimated transportation modes, except walking, assumed access to the nearest road by foot and travel by an immediately available vehicle to the closest facility providing EmONC care. We did not consider district boundaries as barriers to movement; however, we only estimated access to EmONC facilities within the SMGL-supported districts, allowing for movement between districts but not to facilities outside these districts. With ArcGIS Desktop version 10.3.1 (Environmental Systems Research Institute, Redlands, CA), we created a continuous distribution of estimated travel time needed to reach an EmONC facility for each transportation mode and categorized the continuous travel-time raster into 4 incremental 30-minute travel-time zones (0 to 30 minutes, 31 to 60 minutes, 61 to 90 minutes, and 91 to 120 minutes), plus a fifth category for more than 2 hours of travel time (>120 minutes). Instead of using AccessMod's native Zonal Statistics module, we converted the raster into a shapefile of different travel-time zones in ArcGIS version 10.5. We mapped all travel-time zones to reach any EmONC and CEmONC services for each transportation mode.

Combining the travel-time zones with georeferenced village population data, we estimated the number and proportion of WRA with access to EmONC and CEmONC services within each travel-time zone. We obtained the proportion of WRA within a travel-time zone by summing all WRA residing in villages located within each travel-time zone then dividing by the complete enumerated WRA population. We defined “adequate EmONC access” as the ability to reach an EmONC facility within 2 hours of travel time, and “poor EmONC access” as the inability to reach an EmONC facility within 2 hours. We assumed all travel to be from a woman's home to a facility.

We calculated the relative percentage change in the proportions of WRA residing within each travel-time zone and across each transportation mode, by subtracting the baseline percentage from the endline percentage and dividing by the baseline percentage. For the population percentages, *z* scores, based on the normal approximation to the binomial distribution, were used to calculate *P* values.

### Ethical Approval

The study protocol was reviewed and approved by recognized ethics committees in Uganda and complied with Ugandan Ministry of Health procedures for protecting human subjects. This study was reviewed and approved by the U.S. Centers for Disease Control and Prevention's Center for Global Health Human Subject Review Board, which determined that it did not constitute human subjects research.

## RESULTS

At SMGL baseline, substantial differences were noted between the recommended and observed number of EmONC facilities in the 4 SMGL-supported districts (Table 2). Only 10 facilities in a population of over 2 million provided EmONC services, 7 of which provided CEmONC. None of the districts met the recommended benchmark for per-capita EmONC availability. Three districts met the recommended benchmark of at least 1 CEmONC facility at baseline (Kabarole, Kibaale, and Kyenjojo), while Kamwenge had no CEmONC facility.

**TABLE 2.** Recommended and Observed Number of EmONC^a^ and CEmONC Facilities per Capita in SMGL-Supported Districts at Baseline (2012), Phase 1 (2013), and Endline (2016)

	Baseline				Phase 1				Endline			
	Population^a^	Recommended EmONC^b,^^c^	Observed EmONC^c,^^d^	Observed CEmONC^c,^^e^	Population^a^	Recommended EmONC^b^^,^^c^	Observed EmONC^c,^^d^	Observed CEmONC^c,^^e^	Population^f^	Recommended EmONC^b,^^c^	Observed EmONC^d^	Observed CEmONC^c,^^e^
Kabarole	415,600	5	3	3	421,700	5	8	6	456,052	5	8	6
Kibaale	681,300	7	3	3	717,500	8	5	5	818,176	9	7	5
Kamwenge	332,000	4	3	0	339,500	4	5	2	392,501	4	3	3
Kyenjojo	383,600	4	1	1	397,700	4	7	3	428,451	5	8	3
Total	1,812,500	20	10	7	1,876,400	21	25	16	2,095,180	23	26	17

Abbreviations: CEmONC, comprehensive emergency obstetric and neonatal care; EmONC, emergency obstetric and neonatal care; RAMOS, Reproductive Age Mortality Study; SMGL, Saving Mothers, Giving Life; WHO, World Health Organization.

a Estimated from SMGL RAMOS 2013.^19^

b EmONC encompasses facilities performing at least 7 lifesaving interventions within the past 3 months. CEmONC indicates those facilities providing 9 lifesaving interventions in the past 3 months.

c Uses the WHO minimum-recommended number of EmONC and CEmONC per 500,000 population (5 EmONC, including at least 1 CEmONC, per 500,000).

d Observed EmONC includes facilities that may not have provided assisted vaginal delivery in the past 3 months.

e Observed CEmONC includes facilities that may not have provided assisted vaginal delivery in the past 3 months; a few facilities reported shortage of blood in Phase 1 in the previous 3 months but were still classified as CEmONC facilities.

f Estimated from SMGL RAMOS 2017.^19^

Source: SMGL Uganda Health Facility Assessments, 2012, 2013, and 2016.

At baseline, substantial differences were noted between the recommended and observed number of EmONC facilities in the SMGL-supported districts.

SMGL increased the number of facilities performing EmONC across all districts. Most of the increases occurred during SMGL's first year (Phase 1), when the total number of EmONC facilities more than doubled, from 10 to 25 facilities (Table 2). Comparatively fewer changes in the number of EmONC facilities occurred between the conclusion of Phase 1 and endline; an additional 2 EmONC facilities in Kibaale and 1 in Kyenjojo were added, while Kamwenge lost 2 EmONC facilities.

At endline, Kyenjojo and Kabarole exceeded the WHO-recommended number of EmONC facilities, achieving 8 EmONC facilities for over 400,000 people per district. Although Kamwenge attained the WHO benchmarks in Phase 1, it lacked the recommended per-capita number of EmONC facilities at endline. Kibaale, the most populous district, never met the WHO benchmarks for EmONC, despite increasing its total number of EmONC facilities from 5 to 7, with 5 of the 7 providing CEmONC at the conclusion of both Phase 1 and endline.

At endline, Kyenjojo and Kabarole exceeded the WHO-recommended number of EmONC facilities, achieving 8 EmONC facilities for over 400,000 people per district.

Table 3 provides the percentage of estimated WRA population able to reach EmONC and CEmONC within 2 hours or more than 2 hours, by transportation mode, for the 4 districts combined. The greater number of EmONC facilities resulted in significant increases in the proportion of WRA with “adequate” access (within 2 hours) to both EmONC and CEmONC (Table 3).

**TABLE 3.** Estimated Proportion of WRA Within Each Travel-Time Zone in SMGL-Supported Districts in Uganda (2012–2016), by Transportation Mode

Transportation Mode^a^	EmONC Facilities					CEmONC Facilities				
	Baseline, 2012 (%)	Phase 1,2013 (%)	Endline, 2016 (%)	% Change^b^	Sig.Level^c^	Baseline, 2012 (%)	Phase 1, 2013 (%)	Endline, 2016 (%)	% Change^b^	Sig.Level^c^
**Walking, minutes**										
0–30	1.4	2.4	2.6	+88	***	1.2	1.9	1.9	+55	***
31–60	1.5	3.5	3.5	+131	***	1.4	2.4	2.7	+96	***
61–90	2.0	4.0	4.4	+124	***	1.7	3.0	3.1	+89	***
91–120	1.8	4.3	4.5	+152	***	1.5	3.2	3.3	+118	***
≤120	6.7	14.2	15.0	+125	***	5.8	10.5	11.1	+91	***
>120	93.3	85.8	85.0	−9	***	94.2	89.5	88.9	−6	***
**Bicycle, minutes**										
0–30	3.7	6.3	6.5	+77	***	3.4	4.8	5.0	+45	***
31–60	4.0	8.6	8.5	+113	***	3.5	6.6	6.8	+96	***
61–90	4.8	10.4	11.0	+129	***	3.6	7.7	7.9	+118	***
91–120	5.4	10.8	11.6	+115	***	3.8	9.2	9.4	+150	***
≤120	17.9	36.0	37.6	+110	***	14.3	28.2	29.1	+103	***
>120	82.1	64.0	62.4	−24	***	85.7	71.8	70.9	−17	***
**Motorcycle, minutes**										
0–30	11.6	21.3	21.9	+89	***	10.6	16.9	17.2	+62	***
31–60	15.7	22.3	22.5	+44	***	12.2	22.3	23.2	+91	***
61–90	19.7	16.8	16.8	−15	***	15.7	17.6	17.7	+13	***
91–120	14.3	10.7	10.9	−24	***	12.7	12.0	11.7	−7	***
≤120	61.3	71.2	72.1	+18	***	51.1	68.7	69.8	+37	***
>120	38.7	28.8	27.9	−28	***	48.9	31.3	30.2	−38	***
**4-wheeled vehicles, minutes**										
0–30	13.7	24.7	25.4	+85	***	12.3	20.2	20.6	+68	***
31–60	19.4	23.5	23.2	+20	***	15.8	24.1	25.2	+60	***
61–90	20.1	15.1	15.4	−23	***	15.8	16.3	15.9	+1	NS
91–120	11.9	10.2	10.2	−15	***	11.4	10.7	10.6	−7	***
≤120	65.1	73.4	74.1	+14	***	55.2	71.3	72.3	+31	***
>120	34.9	26.6	25.9	−26	***	44.8	28.7	27.7	−38	***

Abbreviations: CEmONC, comprehensive emergency obstetric and newborn care; EmONC, emergency obstetric and newborn care; SMGL, Saving Mothers, Giving Life; Sig. level, Significance level; WRA, women of reproductive age.

a Walking mode includes walking alone. Bicycle mode includes walking to a road and use of a bicycle. Motorized transportation modes (motorcycle, 4-wheeled vehicles) include walking to the road and use of a motorized transportation thereafter.

b Relative % change (% change) is calculated by the formula, ((p2-p1)/p1)*100.

c Asterisks indicate significance level of the % change between baseline and endline, calculated using *z* scores: *** *P*<.01, ** *P*<.05, NS = not significant.

As expected, there were large differences in access between nonmotorized and motorized transportation modes. However, adequate access improved substantially during the SMGL initiative, regardless of the transportation mode. Adequate access to EmONC services by nonmotorized transportation (walking) increased from 6.7% at baseline to 15.0% at endline (125% relative increase) and from 17.9% to 37.6% by bicycle (110% increase). Adequate EmONC access by motorized transport was higher; access to EmONC by motorcycle increased from 61.3% at baseline to 72.1% at endline (17% increase), while access by 4-wheeled vehicle rose from 65.1% to 74.1% (14% increase). Relative increases in the percentage of WRA with adequate access to CEmONC using motorized transport also occurred. Adequate access to CEmONC by motorcycle increased from 51.1% to 69.8% (37% increase), while access by 4-wheeled vehicles increased from 55.2% to 72.3% of WRA (31% increase). Additionally, the percentage of WRA with access to EmONC within 60 minutes or less by motorcycle increased from 27.3% to 44.4% (63% increase) and by 4-wheeled vehicles increased from 33.1% to 48.6% (47% increase).

**FIGURE 2:**
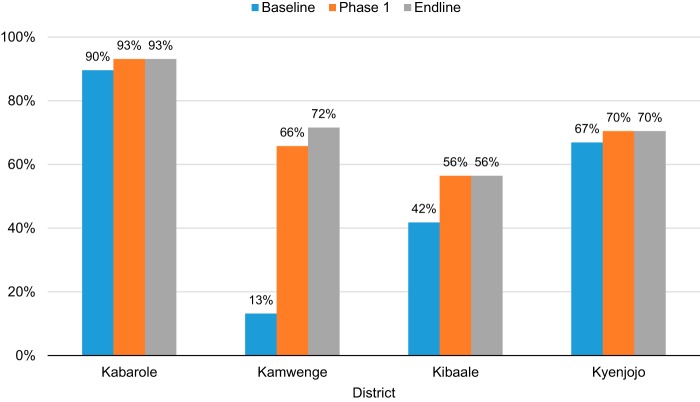
Percentage of Women of Reproductive Age Living Within 2 Hours of CEmONC Facilities by Motorized Transportation and SMGL District Abbreviations: CEmONC, comprehensive emergency obstetric and newborn care; SMGL, Saving Mothers, Giving Life.

When stratified by district, there were substantial differences in the baseline and endline proportions of WRA with adequate access to CEmONC (Figure 2), with similar patterns for EmONC access by district (data not shown). Kabarole, the most urbanized and densely populated district, began SMGL with 89.6% of estimated WRA having adequate access to CEmONC, which increased to 93.1% at endline, a relative increase of about 3%. Conversely, in sparsely populated Kamwenge, where only 13.1% of estimated WRA had adequate access at baseline, the added SMGL-supported facilities increased adequate CEmONC access to 71.6% at endline, a 447% increase. In Kibaale, the proportion of WRA with adequate access to CEmONC was 41.8% at baseline but increased to 56.5% by endline, a 35% increase. In Kyenjojo, adequate CEmONC access increased from 66.9% of WRA at baseline to 70.5% at endline, a relatively modest 5% increase.

In Kamwenge, where only 13.1% of WRA had adequate access at baseline, the added SMGL-supported facilities increased adequate CEmONC access to 71.6% at endline, a 447% increase.

**FIGURE 3:**
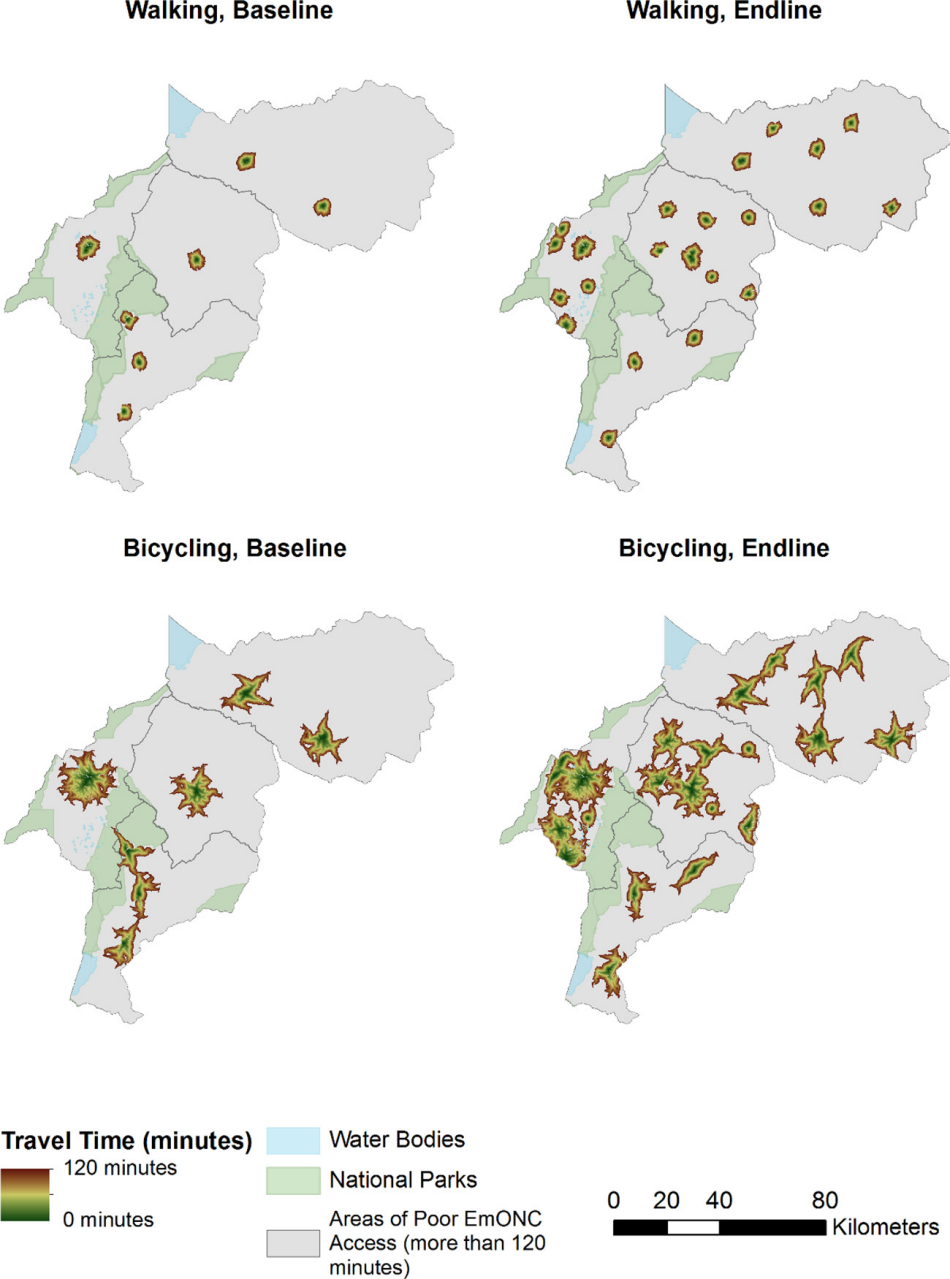
Distribution of Estimated Travel Time to EmONC Facilities, Walking or Bicycling and Walking Abbreviation: EmONC, emergency obstetric and newborn care.

**FIGURE 4:**
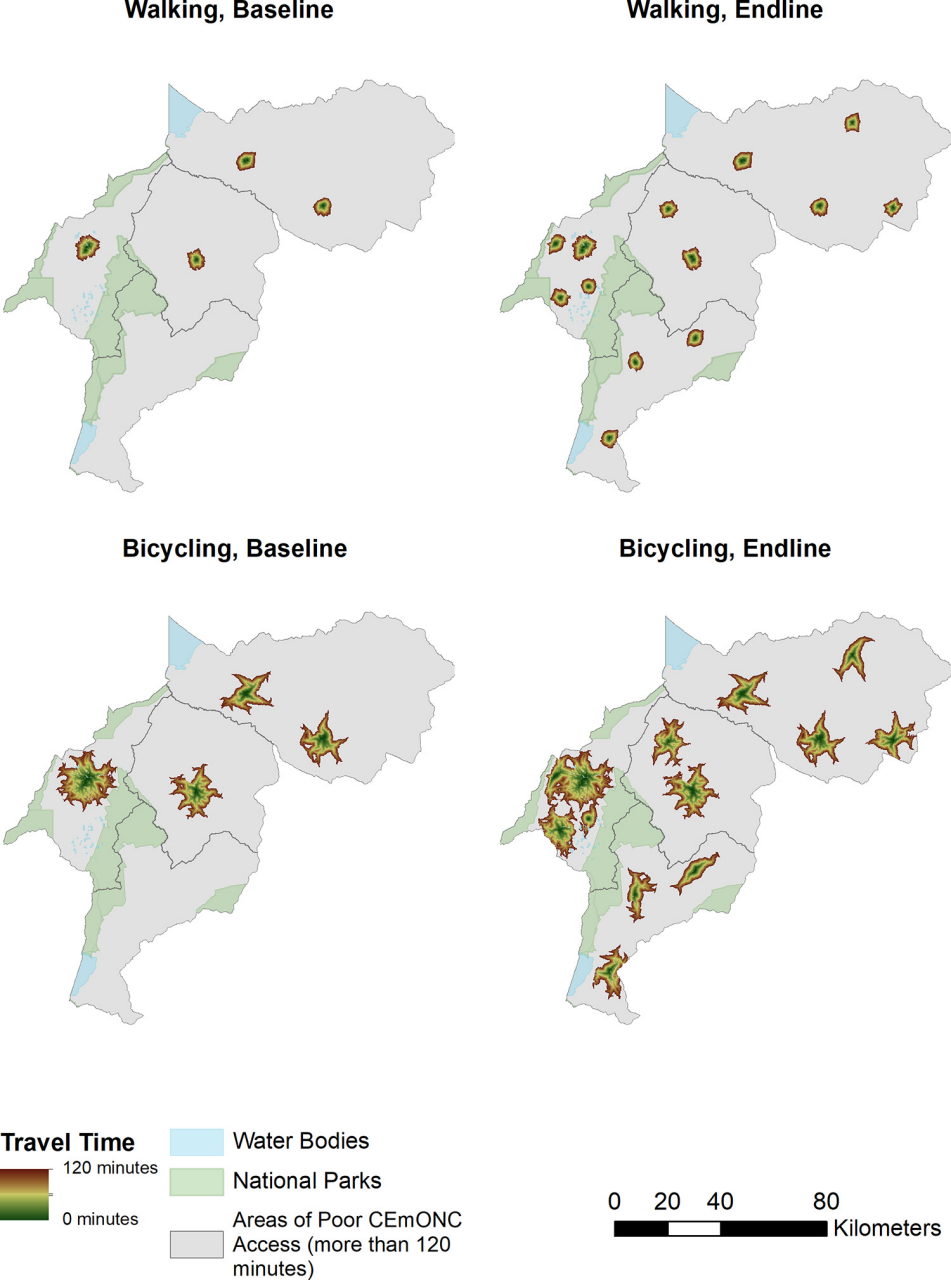
Distribution of Estimated Travel Time to CEmONC Facilities, Walking or Bicycling and Walking Abbreviation: CEmONC, comprehensive emergency obstetric and newborn care.

**FIGURE 5:**
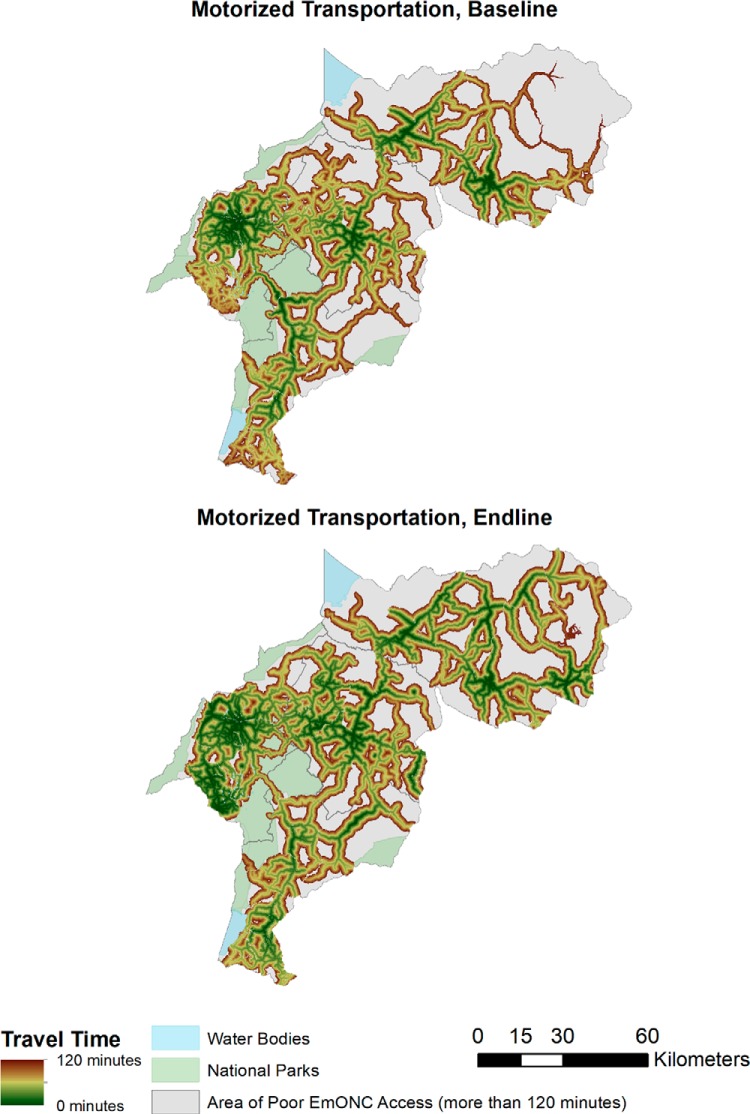
Distribution of Estimated Travel Time to EmONC Facilities Using Motorized Transportation^a^ ^a^ Transportation defined as motorcycles or 4-wheeled vehicles. Abbreviation: EmONC, emergency obstetric and newborn care.

**FIGURE 6:**
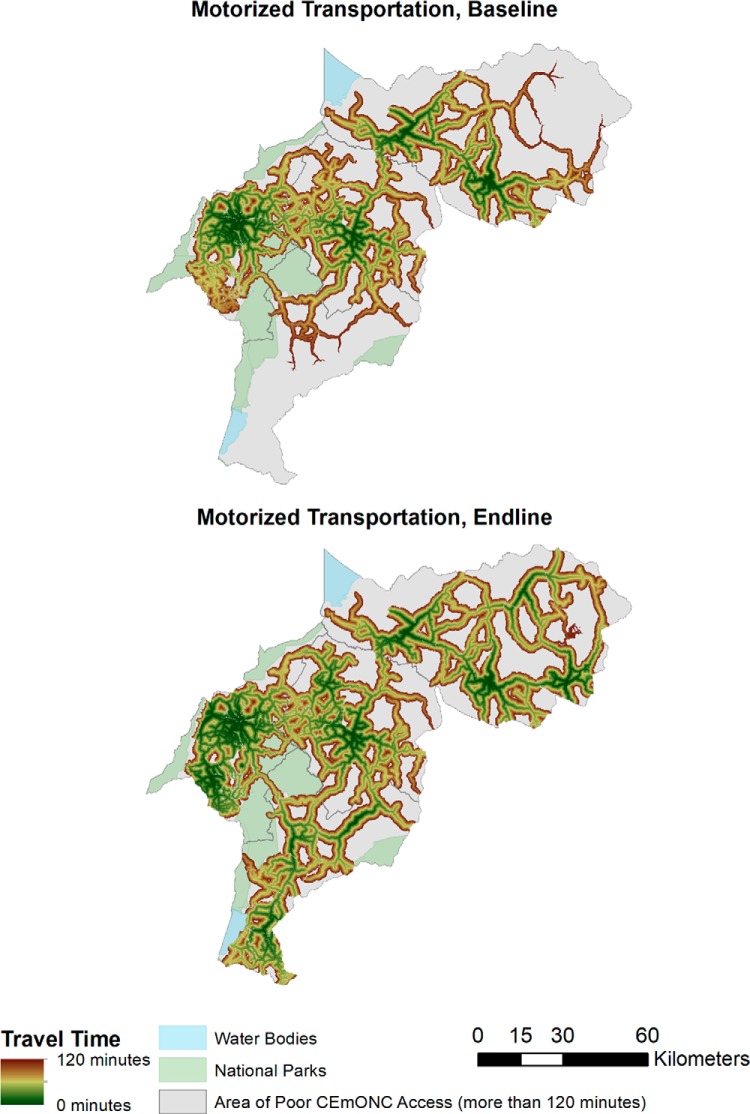
Distribution of Estimated Travel Time to CEmONC Facilities Using Motorized Transportation^a^ ^a^ Transportation defined as motorcycles or 4-wheeled vehicles. Abbreviation: CEmONC, comprehensive emergency obstetric and newborn care.

Maps depicting travel-time zone access to EmONC and CEmONC by nonmotorized (Figure 3 and Figure 4) and motorized (Figure 5 and Figure 6) transportation help visualize access improvements that occurred after the addition of new services supported by SMGL. The maps provide a gradient of the travel time needed for adequate access to EmONC services. Adequate access to EmONC facilities is shown in a green-to-brown gradient, displaying 0 to 30 and 90 to 120 minutes, respectively. Areas outside of this gradient—whether gray, blue, or green—denote poor EmONC access.

At baseline, access to EmONC services was concentrated around major towns in Kabarole, Kyenjojo, and Kibaale, with some additional services along the Kabarole-Kamwenge road (Figure 3 and Figure 5). With the addition of new EmONC services through the SMGL initiative, areas with adequate access appear dispersed across the districts. For nonmotorized transportation, the amount of land covered by the zones of adequate access is small, indicating that the majority of WRA still had poor access in the absence of motorized transport. Adequate access to EmONC by motorized transportation was clearly expanded into new areas of Kamwenge district and central and southern Kibaale districts. Similar geographic patterns were found for CEmONC access (Figure 4 and Figure 6). SMGL upgrades and additions led to more widespread distribution of adequate EmONC and CEmONC access. While the areas with poor EmONC and CEmONC access shrank in most districts, several notable gaps in access by motorized transportation remained, particularly on the northern border of Kibaale, in eastern Kamwenge, in western Kabarole, and on the border between Kabarole and Kamwenge.

## DISCUSSION

Addressing access to care requires a systems approach, including synergistic interventions at the community, facility, and health system levels designed to decrease travel time to care and increase access to motorized transportation. Our travel-time analyses show that the SMGL initiative reduced travel time to EmONC services through a rapid expansion of health facilities able to provide EmONC. The number of facilities providing EmONC and CEmONC services more than doubled from baseline to endline. Two districts met the WHO standard for EmONC, while all 4 districts met the standard of at least 1 CEmONC facility. Overcoverage of EmONC care in Kabarole district and undercoverage in Kamwenge and Kibaale districts left the population of Kibaale and Kamwenge districts with less than the recommended number of EmONC facilities per capita by endline.

The number of facilities providing EmONC and CEmONC services more than doubled from baseline to endline.

A central goal of the SMGL initiative was to ensure that all WRA in each SMGL-supported district had access to EmONC within 2 hours of travel. Despite SMGL's extensive facility upgrade achievements, an estimated one-quarter of WRA continued to have poor access to EmONC care by motorized transportation at the conclusion of the initiative. Because higher-level CEmONC facilities were distributed inequitably due to overconcentration in urban areas, access to CEmONC remained especially uneven across the districts. For example, despite Kyenjojo adding 2 facilities over the course of the SMGL initiative, the new CEmONC facilities were located close to an existing CEmONC facility. This led to only a 5% increase in adequate CEmONC access across the district by motorized transport. In contrast, in Kamwenge district, where access to CEmONC care by motorized transport at baseline was very limited, the addition of 3 geographically distributed CEmONC facilities significantly increased access to CEmONC care (447% increase). Clustering of high-level facilities in urban areas is a problem for increasing access to care to larger geographic areas, unless access to motorized transport is increased.

It is worth noting that our analysis focused only on facilities that could provide the full complement of EmONC signal functions. Numerous facilities in SMGL-supported districts provided partial EmONC, with 4 to 5 signal functions, which did not meet the criteria for full EmONC functionality. Taking all facilities that provide delivery services into account, we found that 18% of WRA had poor motorized access—outside of 2 hours—to any facility that provided deliveries at endline (data not shown). To meet remaining geographic gaps, a combination of efforts to bring partial EmONC facilities to full EmONC capacity, combined with efforts to improve motorized transport access in these still-underserved areas, could increase adequate access for WRA who still had poor access at the conclusion of SMGL. Strategic placement of EmONC facilities in the remaining underserved areas and a focus on equitable distribution could provide a far greater percentage of WRA with adequate access to EmONC care. The results of this analysis provide a geographic outline for future strategically located upgrades to facilities in areas with continued poor access.^26^^,^^27^ Additionally, the SMGL geodatabase could be used to inform other public health efforts in the districts, including immunization campaigns, and placement of other essential services such as family planning and HIV testing.

Strategic placement and equitable distribution of EmONC facilities in the remaining underserved areas could provide a far greater percentage of WRA with adequate access to EmONC care.

Although SMGL did not capture systematic baseline and endline data on the actual transportation used by women in SMGL-supported districts to reach care, there is evidence that the actual use of motorized transport increased. Deliveries in EmONC facilities increased from 28.2% to 41.0% in the SMGL-supported districts in Uganda.^19^ According to exit interviews at EmONC facilities conducted at the conclusion of Phase 1, 90% of women used motorized transportation to reach the EmONC where they delivered.^53^ Additionally, SMGL implementing partners supported the use of motorized transportation through “boda-for-mothers” vouchers—private-service vouchers that included subsidized motorcycle transport—and organized a district ambulance network that included 5 4-by-4 ambulances and 16 eRanger tricycle ambulances at facilities.^20^^,^^21^ Linking ambulances through a district network allowed the closest ambulance to the emergency to be assigned for timely referral of mothers and newborn babies with complications.^53^ The redemption of vouchers increased over the SMGL implementation period, although the voucher supply was reduced in Phase 2 due to interruptions in funding.^54^ Although our geographic models included estimates of adequate access by walking, reaching delivery care by nonmotorized means is clearly not practical. Wider availability of motorized transportation to reach EmONC facilities is necessary to ensure adequate access.

Spatial analyses using GIS have great potential to inform programs and policies in safe motherhood initiatives.^55^ Our study was unique in that we were able to perform travel-time modeling across multiple time points of a multi-year safe motherhood project. The GIS analyses benefited from the project's investments in health systems strengthening at the district level and extensive monitoring and evaluation efforts.

Our travel-time models were based on the most recent data about road network, population, and facility functionality using health facility and reproductive health census data. While we had the opportunity to use direct current population counts by village instead of modeled raster population estimates, publicly available population raster data have been used in other travel-time modeling analyses.^17^^,^^30^^,^^43^^,^^56^^–^^63^

By using open-source programs—such as AccessMod—and advancements in geospatial technologies, it has become easier to perform meaningful actionable travel-time analyses that can be used with monitoring and evaluation programs. With minimum investment in measuring devices, training, and software, district personnel can use existing health information management data or special EmONC assessment studies in combination with population and land cover data to perform travel-time analyses.

### Limitations

This analysis has several limitations. As noted, although we were able to estimate that an increased proportion of WRA had adequate access to EmONC at SMGL endline, the analysis was limited by the lack of data on actual changes in transportation use for women delivering in EmONC facilities. Therefore, we could not assess the relative contributions of decreased distance to care versus greater availability and use of motorized transportation. In addition, the actual proportion of WRA with adequate EmONC access may be lower or higher than our estimates due to assumptions we made. First, we used land and road class speeds that were relatively conservative compared with another recent global analysis.^17^ While lower than the average, the speeds we applied did not account for any variation that might have occurred due to varying road conditions/obstructions or traffic patterns. We estimated travel times for dry road conditions only and could not account for flooding or poor road conditions in rainy seasons. These types of barriers would most likely have reduced speed and lengthened travel time to care, or made travel impossible, as shown in a study conducted in Mozambique.^64^ We assumed that women walk in the most logical path—usually a straight line—from their homes to the nearest road, from where they would access the nearest facility. In real life, women may take various routes to access the nearest road and to travel to the nearest EmONC facility. They may take a longer route if a road or bridge is under repair or if they do not go to the nearest EmONC facility. They may temporarily move away from their home close to their delivery date to live in a family member's home or maternity waiting home near an EmONC facility. Our analysis also did not take into account referrals from lower-level facilities to EmONC facilities.

Geographic inaccessibility is a major barrier to reducing preventable maternal and newborn mortality and morbidity.

Additionally, our estimates did not differentiate between private and public facilities. Access times may be longer for WRA living within an adequate distance to private care but who have to travel further to a public facility. While SMGL-supported private service vouchers with transportation support could augment access to private delivery facilities among women who could otherwise not afford the costs, these vouchers were available only during Phase 1.^20^ Therefore, Phase 1 distance-to-care estimates may be less affected by differential access to private care than Phase 2 estimates.

## CONCLUSION

Our study findings suggest that reducing distance to and increasing optimal distribution of EmONC facilities can increase adequate access to EmONC. Increases in the proportion of WRA with adequate EmONC access due to the SMGL initiative suggest that the SMGL-supported districts made significant advances in bridging the gap in access to timely emergency delivery care. Further gains could be achieved using spatial analyses to strategically estimate placement of EmONC services to reach the greatest number of geographically disadvantaged women, either through the addition of new facilities or upgrading of existing facilities already providing routine obstetric care.

Health care modeling of distance and travel time can help inform the planning of appropriate interventions to overcome spatial disparities in access to maternity care in sub-Saharan Africa. Geographic inaccessibility is a major barrier to countries' efforts to reduce preventable maternal and newborn mortality and morbidity. Periodic assessments of EmONC capabilities, locations, and travel time to EmONC services that take into account actual geographic conditions can enable policy makers and planners to make more informed decisions on the spatial distribution of services and the most effective strategies to improve access.

## Notes

**Peer Reviewed**

**Funding:** Saving Mothers, Giving Life implementation was primarily funded by the Office of the Global AIDS Coordinator, the U.S. Agency for International Development (USAID), Washington, D.C., the Centers for Disease Control and Prevention (CDC), Atlanta, Georgia (Cooperative agreements GPS003057 and GPS002918), Merck for Mothers, and Every Mother Counts. The funding agencies had no influence or control over the content of this article.
